# Connecting high-temperature and low-temperature protein stability and aggregation

**DOI:** 10.1371/journal.pone.0176748

**Published:** 2017-05-04

**Authors:** Mónica Rosa, Christopher J. Roberts, Miguel A. Rodrigues

**Affiliations:** 1 Centro de Química Estrutural, Department of Chemical Engineering, Instituto Superior Técnico, Universidade de Lisboa, Lisboa, Portugal; 2 Department of Chemical & Biomolecular Engineering, University of Delaware, Newark, Delaware, United States of America; Universidad de Granada, SPAIN

## Abstract

Protein aggregation is a long-standing problem for preservation of proteins in both laboratory settings and for commercial biotechnology products. It is well established that heating (cooling) can accelerate (slow) aggregation by populating (depopulating) unfolded or partially unfolded monomer states that are key intermediates in aggregation processes. However, there is a long-standing question of whether the same mechanism(s) that lead to aggregation under high-temperature stress are relevant for low-temperature stress such as in refrigerated or supercooled liquids. This report shows the first direct comparison of “hot” and “cold” aggregation kinetics and folding/unfolding thermodynamics, using bovine hemoglobin as a model system. The results suggest that the same mechanism for non-native aggregation holds from “hot” to “cold” temperatures, with an aggregation temperature-of-maximum-stability slightly below 0°C. This highlights that sub-zero temperatures can induce cold-mediated aggregation, even in the absence of freezing stresses. From a practical perspective, the results suggests the possibility that cold-stress may be a useful alternative to heat-stress for extrapolating predictions of protein shelf life at refrigerated conditions, as well as providing a foundation for more mechanistic studies of cold-stress conditions in future work. A comparison between isochoric and isobaric methods is also briefly discussed.

## Introduction

Non-native protein aggregation is a common challenge associated with processing, handling, and storage of recombinant proteins—particularly those intended as drug products.[1] This is a common concern, as even a small percentage of aggregated molecules can increase the risk of undesired patient immune responses. Aggregation during long-term or short-term cold storage is often difficult to predict from typical “accelerated”conditions.[2,3]

Non-native aggregation refers to the process(es) by which proteins aggregate via partially or fully unfolded (non-native) conformational states. This occurs because the same forces that drive protein folding also drive proteins to form inter-protein contacts that help bury hydrophobic residues, maximize hydrogen bonding and favorable van der Waals contacts, etc.[4,5] For thermal unfolding experiments in practical applications, non-native aggregation often precludes accurate determination of the thermodynamics of protein unfolding—e.g., the free energy of unfolding (ΔG_un_) as a function of temperature (T). As a simple way to see why this is the case, one can consider the Lumry-Eyring model for folding in competition with simplified irreversible processes,[6] or the extended Lumry-Eyring (ELE) model for folding in competition with a series of irreversible aggregation steps.[7,8]

**Fig 1:**
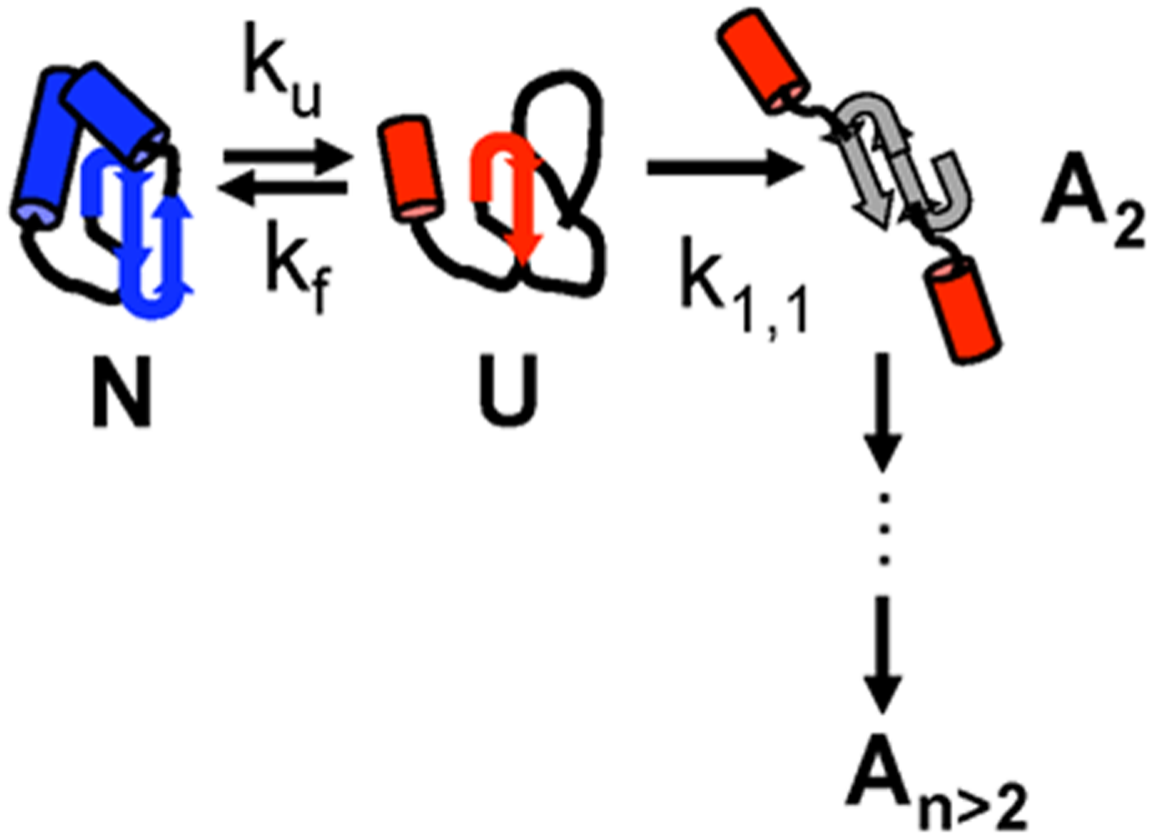
Schematic depiction of folding / unfolding in competition with aggregation. See main text for details.

Fig 1 is a schematic description for two-state protein (un)folding between the monomeric folded or native state (*N*) and an unfolded or partially unfolded state (*U*). This process is in competition with nonnative aggregation (hereafter simply referred to as aggregation) that consumes *U* proteins to create dimers (*A*_2_) and larger aggregates (*A*_n_, with *n* > 2). In Fig 1, *k*_*f*_, *k*_*u*_, and *k*_*1*,*1*_ denote rate coefficients for folding, unfolding, and dimerization, respectively. Additional rate coefficients for aggregation may also be relevant if large soluble aggregates form readily.[9–11] At elevated *T*, the unfolding equilibrium shifts towards the unfolded or partially unfolded state. Therefore, the net concentration of *U* molecules increases at fixed overall protein concentration, although the concentration of partially unfolded molecules may still be too low to measure directly until much higher temperatures.[7] The folding “reaction” is reversible (but not necessarily in equilibrium); however, aggregation is irreversible and this causes *U* to be consumed. For many conditions of practical interest, folding is much faster than aggregation (k_f_ >> k_1,1_). Therefore, the fraction of monomers that exist as *U* is determined by the thermodynamics of unfolding. This follows because as soon as a *U* molecule is consumed by an aggregation event it is rapidly repopulated by the fast *N*–*U* dynamic re-equilibration.[8,10,12].

With this in mind, it is useful to recall the seminal work by a number of researchers that showed that proteins can undergo both “hot” and “cold” unfolding.[13] Thermodynamically, this is a natural consequence of the fact that the enthalpy of unfolding (*ΔH*_*un*_) depends strongly on *T*, or equivalently that the heat capacity of unfolding (*ΔC*_*p*_) is large (~ a few kcal mol^-1^K^-1^) for most proteins that fold spontaneously. This can be seen mathematically from the Gibbs-Helmholtz relationship,[14]

$$\Delta G_{un}\left(T\right)=\Delta H_{0}\left(1-\frac{T}{T_{0}}\right)+\Delta C_{P}\left(T-T_{0}\right)-\Delta C_{P}Tln\left(\frac{T}{T_{0}}\right)$$ (1)

where *T*_*0*_ is the temperature at which Δ*G*_*un*_ = 0, Δ*H*_*0*_ is the value of Δ*H*_*un*_ at *T*_*0*_, and Δ*H*_*un*_(*T*) is given by Δ*H*_*0 +*_ Δ*C*_*p*_(*T-T*_*0*_). By convention, *T*_*0*_ is usually taken as the “hot” temperature at which fifty percent of the protein monomers are in the *N* state (on average), and fifty percent are the *U* state at a given moment in time.[13,14] *T*_0_ is sometimes denoted as the “melting” temperature, or more accurately it represents the temperature for the midpoint of the unfolding transition, *T*_*M*_. For single-domain proteins, the fraction of protein monomers that are in the (partly) unfolded state is denoted by *f*_*un*,_ for a given temperature, pressure, and solution composition

$$f_{un}=K{\left(1+K\right)}^{-1}$$ (2)

with $K=e^{-\Delta G_{un}/RT}$, and therefore *f*_*un*_ = 0.5 at *T*_*0*_.

It follows from these arguments, and the van’t Hoff relation, that *f*_*un*_ has a minimum at the temperature for ΔH_un_ = 0 (denoted by *T*_*H*_ in the nomenclature of Becktel and Schellman[14]). Therefore, if aggregation is promoted by increasing the concentration of unfolded or partially unfolded monomers, then aggregation rates should scale with *f*_*un*_ raised to a power greater than one.[7,12,15] In the ELE model, this is represented by the following relationship between the observed rate coefficient for aggregation (*k*_*obs*_) and the intrinsic rate coefficient for dimerization of unfolded monomers (*k*_*1*,*1*_); both *k*_*1*,*1*_ and *f*_*un*_ depend on temperature,[7]

$$k_{obs}\left(T\right)=\lambda k_{1,1}f_{un}^{2}$$ (3)

In Eq 3, λ is a coefficient that depends on whether aggregates grow significantly beyond dimers, and if so then whether the aggregates remain soluble. With the exception of cases when aggregates grow to become large soluble species composed of tens to hundreds of proteins per aggregate, λ is of order 1, with typical values near 2.[7,8,11,12,16,17]

Based on these considerations, it was hypothesized previously that aggregation might also be accelerated by cooling below *T*_*H*_.[8] However, that was not found experimentally because aggregation rates could not be quantified below 0°C in those cases. As a result, prior work showed non-Arrhenius behavior that suggested aggregation might accelerate if one could cool below 0°C, but aggregation rates were not measured below refrigerated conditions.[17]

**Fig 2:**
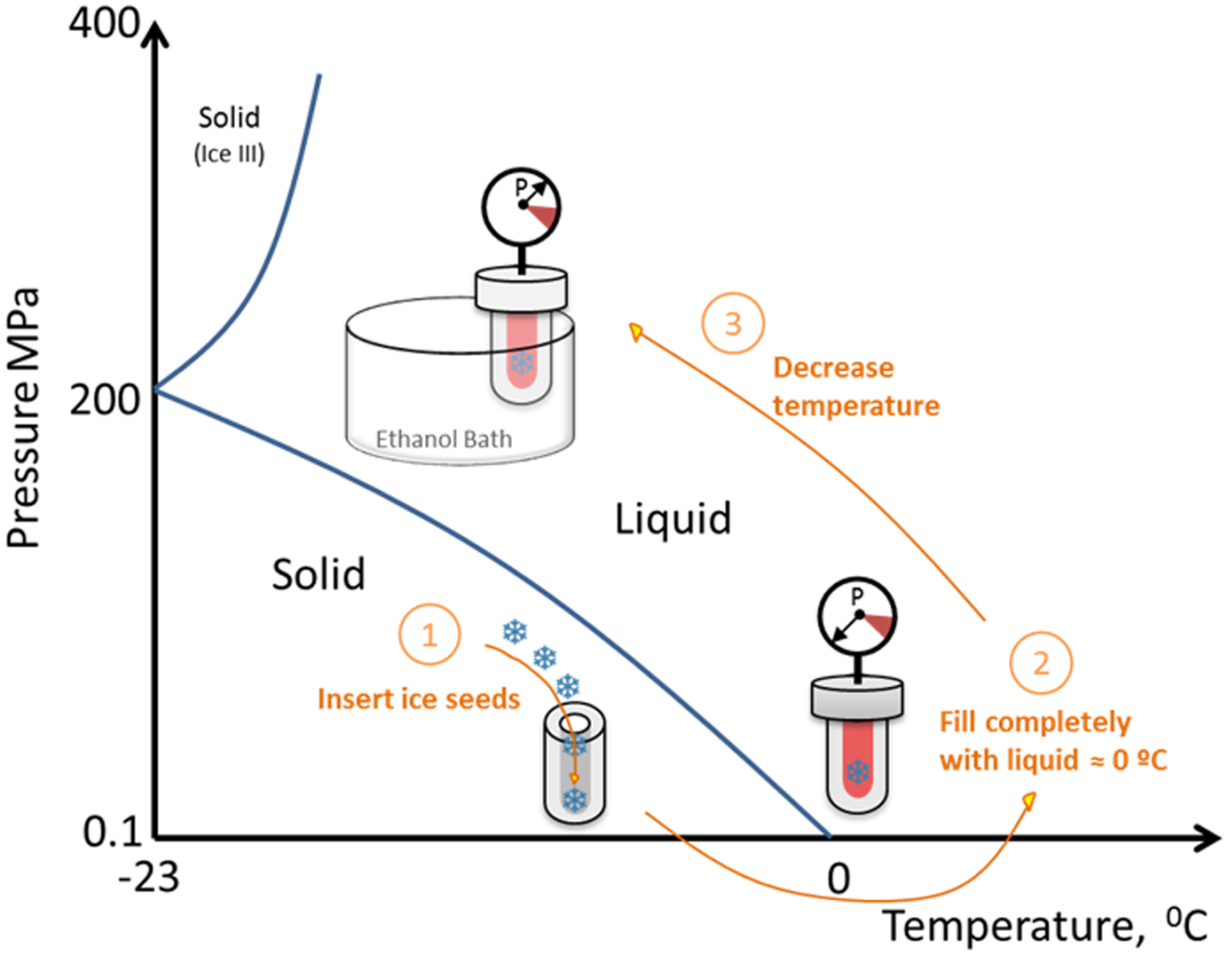
Isochoric method for protein aggregation studies below 0°C in supercooled liquid states. The ice seeds (step 1) provide a natural piston when the reactor is completely full of liquid (step 2); as temperature is lowered the low-density ice attempts to grow, but therefore compresses the solution (step 3); this raises the pressure and suppresses the freezing line, thereby enabling the solution to effectively avoid freezing.

More recent results with bovine hemoglobin (BHb) showed that BHb does undergo cold unfolding, and aggregation rates do increase with increasing cooling into sub-zero liquid states accessed by a novel isochoric cooling method (see Fig 2).[17] This method enables one to maintain the liquid as an equilibrium state below 0°C, by allowing the system pressure to rise. Pressure can also influence the unfolding equilibrium.[18] However, the rise in pressure was found previously to be a secondary effect for hemoglobin, because the temperature of cold denaturation (where 50% of the protein is unfolded) is reached much earlier due to cooling than the pressures required for causing significant unfolding.[17]

Recent work that focused on protein-folding thermodynamics suggests that the two-state approximation (e.g., as used in Eq 2) is too simplified, and should not be an effective description of how the ensemble of protein conformational states changes with temperature and solution conditions.[19] A more compelling test of the effectiveness of simple models such as the ELE aggregation model would then be to show whether both high-temperature and low-temperature aggregation rates can be captured by the same model parameters, and whether “cold” aggregation rates can be predicted by “hot” aggregation rates, or vice versa. In order for such predictions to be successful, the two-state approximation would need to hold reasonably well for at least the aggregation-prone domain of a given protein, and the intrinsic aggregation mechanisms would need to not change with *T*. To the best of our knowledge, no report has shown that one can equivalently accelerate non-native aggregation in solution for a given protein with both heating and cooling, or that this can be quantitatively predicted using only “hot” or “cold” aggregation rate data along with unfolding thermodynamics.

This report focuses on an experimental test of the hypothesis of a common mechanism for “hot” and “cold” aggregation, using BHb as a model system. It combines new thermodynamic and kinetic data from high temperature conditions with published results from low temperatures, to show that a common unfolding-mediated pathway for BHb is able to qualitatively and quantitatively explain both “hot” and “cold” aggregation. This suggests it may be worth considering alternative, “cold acceleration” for assessing protein shelf life, and as an approach to separately interrogate the stresses of “cold” from those due to freezing (e.g., freeze concentration, pH changes due to buffer precipitation, etc.). Finally, the fundamental limitations of temperature-accelerated aggregation experiments for prediction of long-term stability of protein solutions are discussed from a practical perspective, as well as the choice of isochoric versus isobaric methods for reliably accessing conditions below 0°C for stability measurements.

## Materials and methods

BHb was obtained from Sigma Aldrich (US) (reference H2500) and used without further purification. The buffer used for protein studies was phosphate buffer (PB) 0,067 mol L^-1^ at pH = 7.4, analytical grade buffer salts were obtained from Sigma Aldrich.

### Isothermal aggregation at low temperatures by isochoric method

The aggregation experiments were carried in high-pressure reactors (HIP MS-16, USA) with 6 cm^3^ volume. Freezing is hindered when temperature is lowered at constant volume because the density of ice is smaller than liquid water in the range of pressure of this study. Pressure will rise as temperature is lowered following the negative slope of water’s solid-liquid equilibrium. However, uncontrolled nucleation would result in abrupt pressure variation. To prevent this, 0.2 g of water were frozen inside the reactor (1h at -20°C) to provide ice seeds before loading the BHb solution. BHb solutions were then loaded in the reactors carefully avoiding formation of air bubbles, and placed in a controlled temperature ethanol bath cooled by a Haake, type EK 12 immersion cooler (Karlsruhe, Germany). The aggregation curves were determined from samples removed at different times.

The integrity of the isochoric freezing system was confirmed by using a pressure sensor (PX603 from Omega, UK).

### Isothermal aggregation at high temperatures

BHb solutions were prepared for isothermal kinetic studies at a concentration of 1.0 g.L^-1^ (C_0_) in phosphate buffer pH 7.4. All solutions were filtered with a 0,2 μm PVDF membrane, to remove particles, before run start. The BHb solution was then loaded (1 ml) into 2 ml HPLC vials hermetically sealed with HPLC screw caps. Samples were incubated in duplicate for selected times and temperatures to provide a time course for monitoring aggregation kinetics. Isothermal conditions were maintained using a water bath (±0.2°C). The temperatures selected were 23, 26, 31 and 35°C. Samples were removed at designated times throughout aggregation and quenched for at least 5 min in an ice-water bath to arrest aggregation. Samples were visually inspected and those samples with visible haze or particles present were centrifuged at 10,000 g for a minimum of 3 min; the supernatant was collected for further analysis. Each aggregation isotherm was determined by three independent experiments in 3 different days using fresh BHb solutions. For each time, the aggregated fraction resulting from the 6 samples (3 independent curves with duplicate vials) was averaged.

### Size- exclusion chromatography (SEC)

Samples were analyzed for monomer loss and soluble aggregates using SEC. In the context of this work the term “monomer” is a supra molecular characterization (monomer or polymer) and should not be confused with the molecular structure of the molecule. BHb is constituted by four subunits and therefore, from the molecular perspective, our monomer is actually a tetramer. The SEC procedure used a Jasco HPLC-system apparatus equipped with a refractive index detector (RI-2031, Jasco, Japan) and a diode array detector (MD-2010, Jasco, Japan), set at 280 nm. Monomer concentration of BHb was determined by SEC as a function of incubation time for a given initial protein concentration, using a Waters Ultrahydrogel 500 mm and 1000 mm size-exclusion column. The injection volume was 100 μL. The mobile phase (isocratic) was a 50 mM sodium phosphate pH 7.0 with 600 mM sodium chloride. The fraction of monomer remaining is m = M/C_0_, with M is the monomer concentration and C_0_ is the initial concentration of protein.

## Results and discussion

It is common to monitor and quantify aggregation rates in terms of either loss of parent monomer, or the change in surrogate quantities such as weight-average molecular weight or effective diffusion coefficient. The former is usually quantified with chromatography, field-flow fractionation, or analytical ultracentrifugation. The latter are typically measured with static or dynamic light scattering, respectively, and are most useful when aggregates remain small and soluble so that higher-order scattering effects are not problematic.[20] In the present case, all aggregates were essentially insoluble (visible haze and cloudy samples immediately upon any measurable monomer loss) and therefore chromatography of the supernatant after sample centrifugation was the natural choice for quantifying aggregation rates.[8]

3A shows monomer loss profiles (from SEC, see Methods) versus incubation time for BHb at a series of *T* values (room temperature and higher). data are reasonably well described by a simple mass-action kinetic model such as used previously[17]

$$\frac{dm}{dt}=-k_{obs}m^{\alpha }$$ (4)

where *m* is the monomer concentration divided by its initial concentration, and α is the apparent reaction order; in this case α = 2, consistent with the ELE model noted above.^4^ No soluble aggregates were detected by chromatography for any condition (data not shown), which is also consistent with the ELE model with a value of λ = 2 and α = 2. Mechanistically, this corresponds to a case where dimerization is rate-limiting, and aggregates are essentially insoluble once they grow beyond a dimer.[7,12]

**Fig 3:**
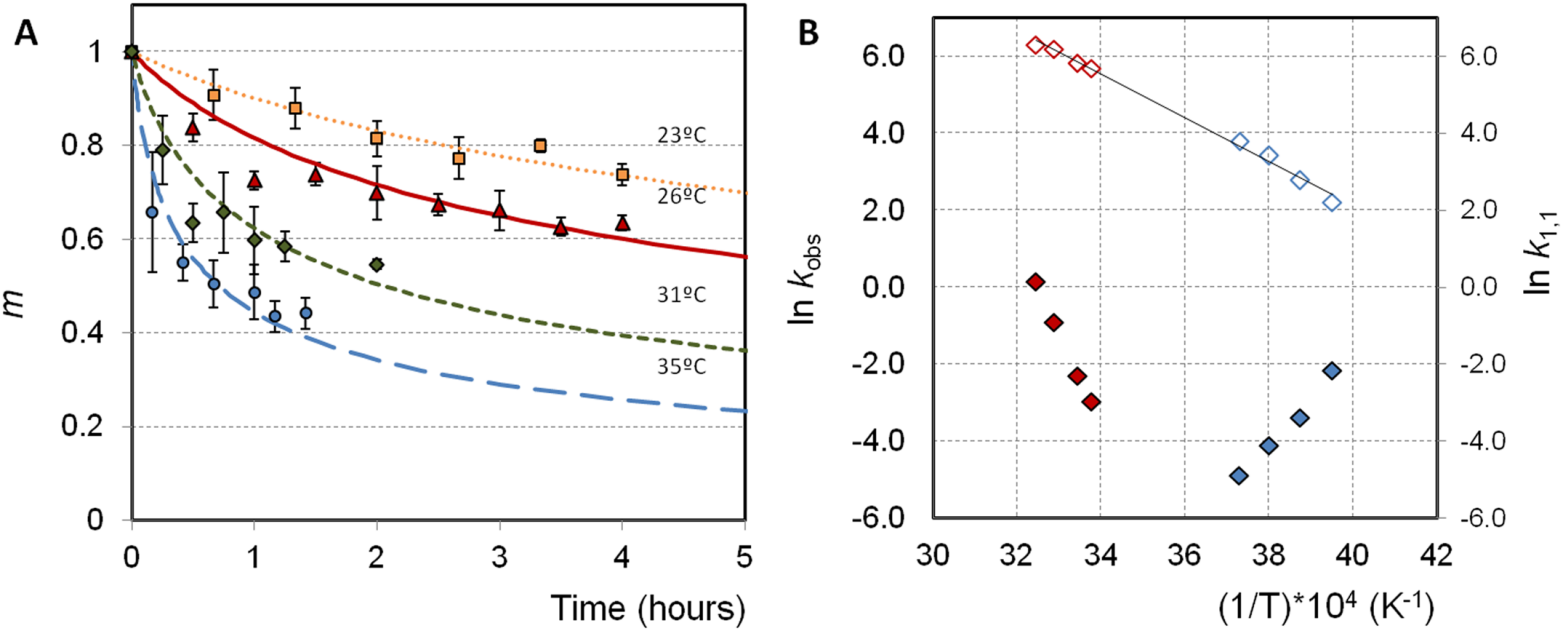
BHb aggregation kinetics. (A) Isothermal aggregation of BHb in phosphate buffer pH 7.4 at different temperatures from 23°C to 35°C and corresponding fitted aggregation curves (see Methods). (B) Temperature dependence of *k*_*obs*_ (closed symbols) and *k*_*1*,*1*_ (open symbols) for BHb spanning from high temperatures (red symbols) to low temperatures (blue symbols). The solid line is an Arrhenius fit to Eq 4 (*E*_*a*_ = 11.4 ± 1.9 kcal mol^-1^ and corresponding *k*_*1*,*1*_ (*T*_*ref*_) from Table 1). The dashed line is an Arrhenius fit to Eq 5 (*E*_*a*_ = 11.3 ± 0.8 kcal mol^-1^ and ln k_1,1_(*T*_*ref*_) = 5.66 ± 0.14). The units of *k*_*obs*_ and *k*_*1*,*1*_ are L g^-1^ h^-1^, to be consistent with previous work.[17].

Fig 3A demonstrates that aggregation rates increase with increasing temperature, which is the canonical expectation for protein solutions. The fitted *k*_obs_ values are given in Table 1, and shown in Fig 3B along with *k*_*obs*_ values from previous work at lower temperatures [17]. Contrary to common expectations, the results in Fig 3B show that BHb aggregation is accelerated by both heating and by cooling. A minimum value for *k*_*obs*_ can be anticipated from the filled symbols in Fig 3B, within the temperature range of -5°C to 20°C. This may correspond to the maximum stability temperature towards unfolding as expressed by Eq 1, which was fitted previously for BHb, with a maximum ΔG_un_ at 11°C [17]. The Gibbs-Helmholtz relationship anticipates the fraction of unfolded molecules will increase exponentially as temperature increases or decreases from the temperature of maximum stability. To test whether this hot and cold aggregation is consistent with a common unfolding-mediated mechanism, the experimental *k*_*obs*_ were combined with experimental values[17] for *f*_*un*_ for each temperature. Using Eq 3, the intrinsic aggregation rate coefficient (*k*_*1*,*1*_) was back-calculated for each temperature, and the values of *k*_*1*,*1*_ are overlaid as open symbols in Fig 3B. In contrast to *k*_*obs*_, the values of *k*_*1*,*1*_ increase monotonically with increasing temperature in the full range of temperature experimented, also shown in Table 1.

**Table 1:** Fitted values for *k*_*ob*s_ and the Normalized Root-Mean-Square Standard Error (NRMSE) associated with each curve. Values for *f*_*un*_ = *f*_*R*_ were calculated from Eq 1 based on the unfolding thermodynamics of BHb reported in Rosa et al.[17] The values for *k*_*1*,*1*_ follow from Eq 3.

Temperature (°C)	*f*_R_ = *f*_*un*_	*k*_11_ (h^-1^)	*k*_obs_ (h^-1^)	NRMSE
-20	0.113	9.1	0.116^(a)^	0.09
-15	0.046	16.1	0.034^(a)^	0.16
-10	0.023	30.9	0.016^(a)^	0.18
-5	0.013	44.6	0.008^(a)^	0.13
23	0.0132	298	0.0520	0.07
26	0.0170	340	0.0984	0.09
31	0.0285	491	0.399	0.20
35	0.0460	553	1.17	0.03

^(a)^from ref. [17].

Physically, *k*_*1*,*1*_ represents the nucleation or dimerization rate coefficient for (partly) unfolded BHb monomers. Previous work with bovine G-CSF found that *k*_*1*,*1*_ followed an Arrhenius temperature dependence. The solid line in Fig 3B is a fit to an Arrhenius form for *k*_*1*,*1*_ of BHb,

$$\text{ln}k_{1,1}\left(T\right)=\text{ln}k_{1,1}\left(T_{ref}\right)-\frac{E_{a}}{R}\left(T^{-1}-T_{ref}^{-1}\right)$$ (5)

where *E*_*a*_ is the activation energy, *T*_*ref*_ is an arbitrarily chosen reference temperature, and *k*_*1*,*1*_(*T*_*ref*_) is the value of *k*_*1*,*1*_ at *T*_*ref*_. Using *T*_*ref*_ = 23°C and fitting *E*_*a*_ and ln *k*_*1*,*1*_(*T*_*ref*_) gives *E*_*a*_ = 11.3 ± 0.8 kcal mol^-1^ and ln *k*_*1*,*1*_(*T*_*ref*_) = 5.66 ± 0.14.

**Fig 4:**
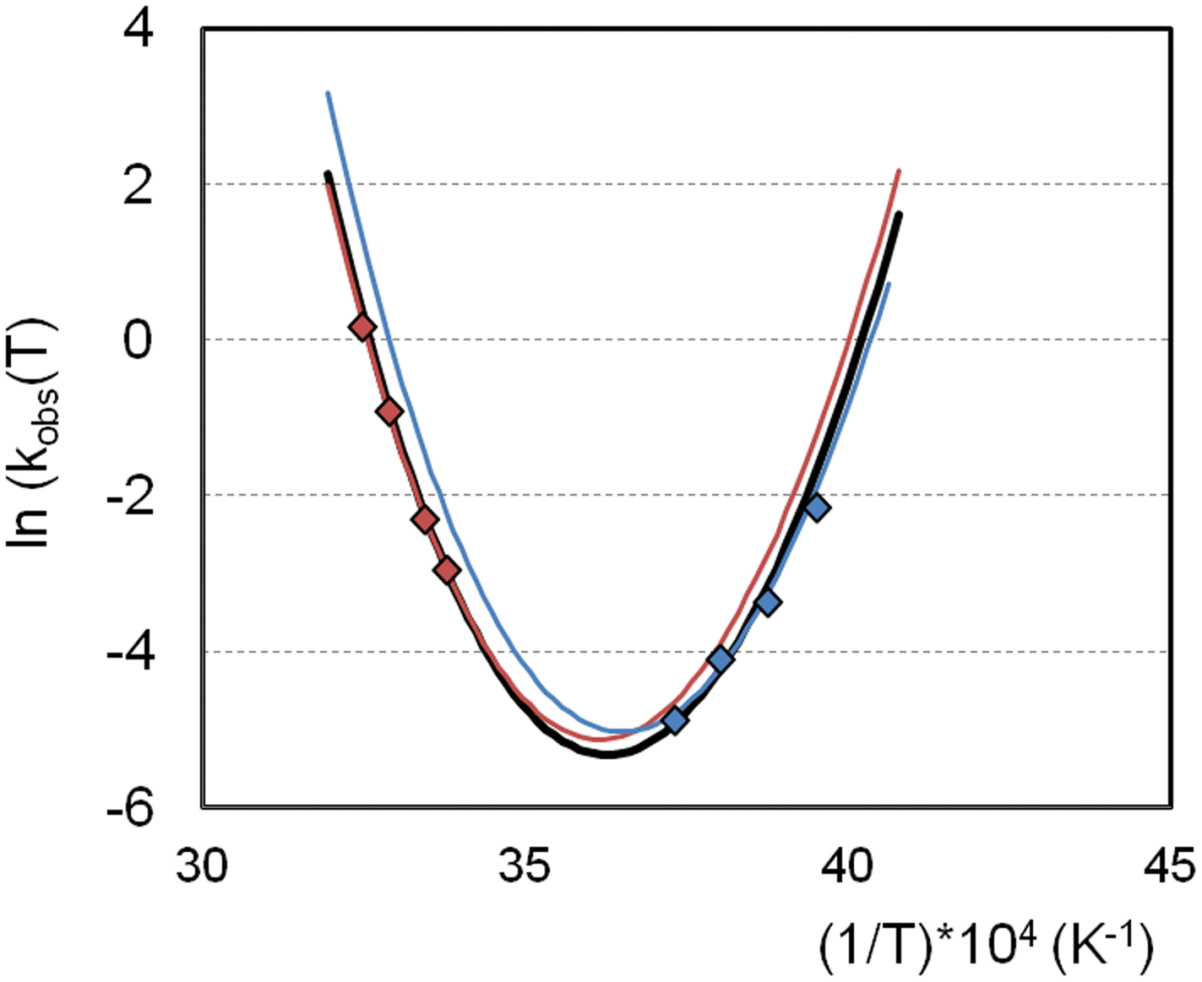
Prediction of the rate coefficient for BHb aggregation (*k*_*obs*_). The lines are the predictions from Eq 6 using the regressed parameters from Eq 4 (see also, main text). Three curves were calculated: in red using *E*_*a*_ obtained from the experimental data of the hot side only (*E*_*a*_ = 9.8 ± 2.0 kcal/mol), in blue using the experimental data of the cold side only (*E*_*a*_ = 13.3 ± 3.2 kcal/mol, using as T_ref_ = -5°C and corresponding and *k*_*1*,*1*_(*T*_*ref*_) from Table 1) and in black using *E*_*a*_ obtained from experimental data from both sides (as shown in Fig 3B).

Eq 5 can be combined analytically with Eq 3 and *f*_*un*_(*T*) to provide predicted values of *k*_*obs*_ at arbitrary temperatures via

$$\text{ln}k_{obs}=\text{ln}k_{ref}-\;\frac{E_{a}}{R}\left(T^{-1}-T_{ref}^{-1}\right)+2\text{ln}\left[\frac{f_{un}}{f_{un}\left(T_{ref}\right)}\right]$$ (6)

where k_ref_ and *f*_*un*_(*T*_*ref*_) are the values of *k*_*obs*_ and *f*_*un*_, respectively, evaluated at *T*_*ref*_. If the same intrinsic aggregation mechanism holds quantitatively for aggregation of BHb at both “cold” and “hot” conditions, then the curve predicted from Eq 6 with the fit to Eq 5 should provide an accurate description of the full *k*_*obs*_(*T*) profile. The black line in Fig 4 shows that there is excellent agreement between the experimental data and the curve from Eq 6. The red and blue lines in Fig 4 show that equivalently accurate predictions can also be obtained by using only experimental data from the “hot conditions” or “cold conditions”, respectively, when fitting and obtaining parameters values for *E*_*a*_ and *k*_*1*,*1*_(*T*_*ref*_) using only the “hot” or “cold” data. Therefore, one can use the hot (cold) conditions to accurately predict aggregation rates at cold (hot) conditions for BHb.

The results show pronounced non-Arrhenius behavior when viewed over the full temperature range, with a minimum-aggregation-rate at slightly below 0°C, which is consistent with the temperature-of-maximum stability (T_MS_) of BHb reported previously [17]. To the best of our knowledge, this is the first time both “hot” and “cold” aggregation have been demonstrated for the same protein and found to follow directly from the same mechanism. Interestingly, the value of the intrinsic aggregation activation energy (*E*_*a*_) for BHb aggregation is relatively low (~ 10 kcal/mol) when compared to that for *k*_*obs*_ at temperatures far above or below T_MS_, consistent with what was observed previously for bovine G-CSF.[8] This is also consistent with other model proteins that show negative activation enthalpies for *k*_*1*,*1*_ or its analogues,[21] and is consistent with the view that there are large entropic barriers for nucleating or initiating aggregates from unfolded proteins.[4,21,22] Therefore, while the overall or measured rate coefficient (*k*_*obs*_) involves contributions from both unfolding/refolding and inter-protein assembly steps, the major changes in overall aggregation rates due to changes in temperature appear to be dominated by changes in unfolding thermodynamics.

The results also illustrate a general concern that linear Arrhenius extrapolation of classical accelerated stability experiments (such as those in Figs 1 and 2A) can grossly overestimate the low-temperature stability of protein solutions.^11,12^ Often, such studies are conducted at temperatures well above room temperature (~ 40°C or higher) because that is necessary to accelerate aggregation rates to reasonable time scales (~ hours to weeks). Indeed, the practical utility of such high-temperature stability studies has been raised frequently.[2,3,16,22–24] The results here offer an alternative, and perhaps more encouraging perspective. If one is interested in stability at cold temperatures (e.g., under refrigeration), one can perform accelerated stability studies at cold(er) temperatures. This may eliminate the need for extrapolating the results over large temperature ranges, and thereby minimize the impact of non-Arrhenius behavior for *k*_*obs*_.

However, in practice, this may require more sophisticated experimental approaches to allow *k*_*obs*_ to be measured while avoiding water freezing.[17] Supercooled liquid states are readily accessed for significant periods of time during processes such as commercial lyophilization, even after water partially crystallizes in the presence of solutes that greatly suppress the freezing point.[25,26] In the present case, this was accomplished without adding cosolutes, by using an isochoric method, which has the necessity of raising the pressure. Nevertheless, the results show an excellent agreement despite the fact that the impact of pressure on the unfolding equilibrium is neglected in the analysis above.

It is anticipated that an isochoric approach could be useful for a range of proteins, because lowering the temperature to -20°C (for example) is expected to bring most mesophilic proteins or their subdomains to approximately 30°C to 50°C below their respective T_H_ values, while the increases in pressure may also help to accelerate aggregation by shifting the F-U equilibrium. However, at much higher pressures aggregates can be destabilized and aggregation would be slowed or even reversed.[27,28] In general, cooling below 0°C is expected to produce a large impact on the unfolding equilibrium of mesophilic proteins. Conversely, the hydrostatic pressure required to significantly unfold a protein is difficult to predict, but relates intrinsically to the change in partial molar volume of unfolding (Δ*v*_un_) via

$${\left(\frac{\partial \Delta G_{un}}{\partial p}\right)}_{T,c_{j}}=\Delta v_{un}=v_{U}-v_{F}$$ (7)

**Fig 5:**
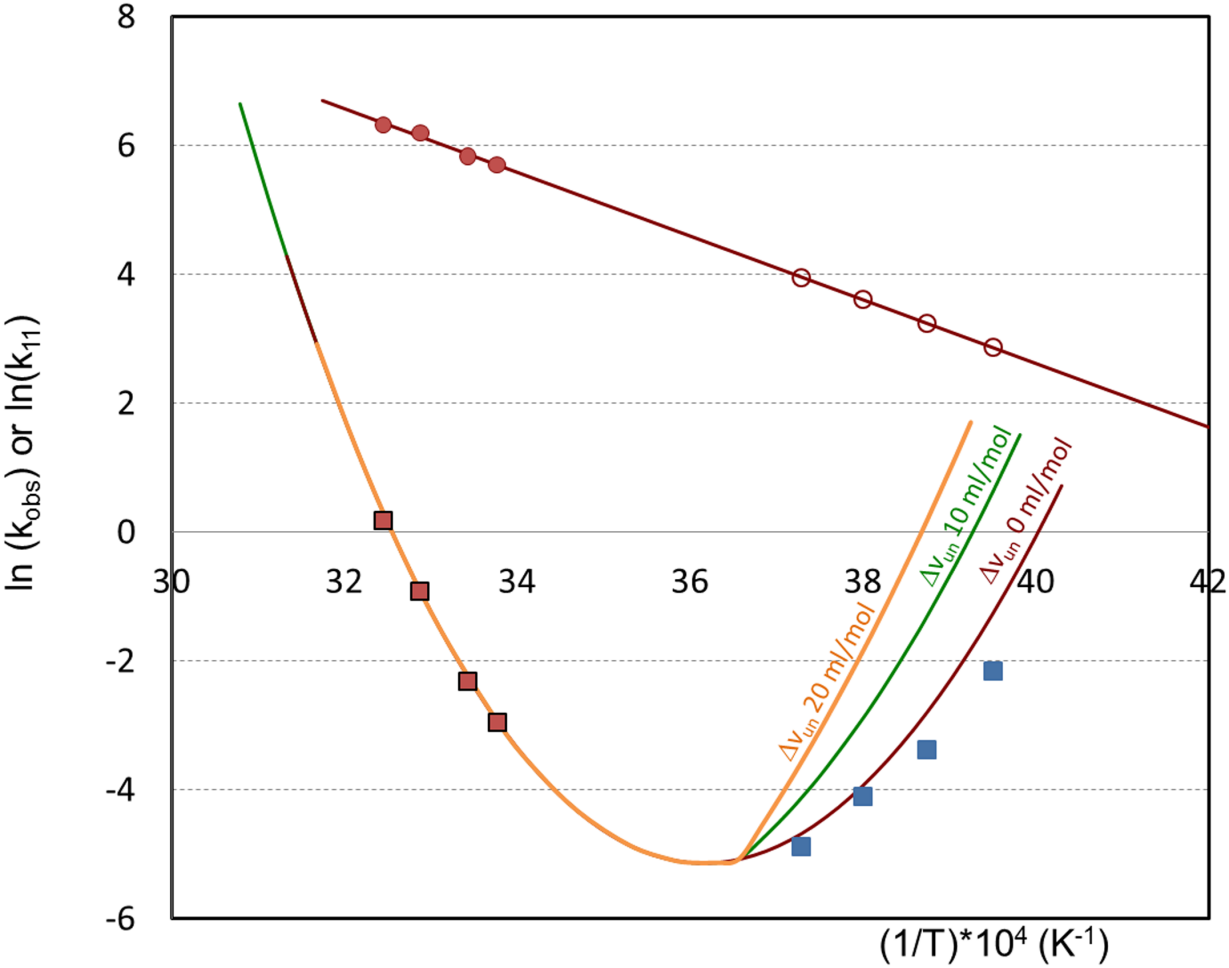
Illustration of the potential influence of BHb volume variation of unfolding (Δ*v*_un_) on its aggregation kinetic constant k_obs_. The k_11_ values (circles) were estimated for low temperature by linearly extrapolating the k_11_ from the high temperature data (at normal pressure). The calculated values of k_obs_ were estimated (curves) for different Δ*v*_un_ (0, 10 and 20 ml/mol) considering the impact of pressure (under isochoric cooling) on the Δ*G*_un_. Experimental k_obs_ are represented by squares.

The value of Δ*v*_un_ for BHb is not known, but globular proteins can have small negative Δ*v*_un_ values, and that is consistent with the agreement of the high-T and low-T results in Figs 3B and 4. If Δ*v*_un_ was large, one would expect the temperature extrapolation inherent in the analysis in Fig 4 to not be quantitatively accurate. Nonetheless, the impact of Δ*v*_un_, on BHb aggregation rates (k_obs_,) may still be illustrated by considering the pressure contribution to BHb unfolding, as shown in Fig 5. The results in Fig 5 are based on recalculating the Δ*G*_*un*_ values and corresponding *f*_*un*_ values using the Gibbs Helmholtz expression Δ*G*_*un*_ as a function of (*p*,*T*), shown below, instead of the previous simplification (Eq 1) for constant pressure:

$$\Delta G_{un}\left(p,T\right)=\Delta H_{0}\left(1-\frac{T}{T_{0}}\right)+\Delta C_{P}\left(T-T_{0}\right)-\Delta C_{P}Tln\left(\frac{T}{T_{0}}\right)+\Delta v_{un}\left(p-p_{0}\right)+\frac{1}{2}\Delta \beta {\left(p-p_{0}\right)}^{2}+\Delta \alpha \left(p-p_{0}\right)\left(T-T_{0}\right)$$ (8)

where beta is the compressibility and alpha the thermal expansivity factor, which were neglected for the illustrative calculation presented in Fig 5. The term Δ*v*_un_Δ*p* was calculated considering that the isochoric cooling follows water’s solid-liquid equilibrium (*p*,*T*). The *k*_obs_ values shown in Fig 5 were therefore calculated by Eq 3, using the k_11_ value obtained from Eq 5 using the correlation of the hot data exclusively, which are at atmospheric pressure (with *E*_*a*_ = 9.8 ± 2.0 kcal/mol).

Overall, Fig 5 shows that calculated k_obs_ correlate better with experimental values when Δ*v*_un_ is close to zero, demonstrating that pressure effects can be reasonably disregarded for BHb. On the other hand, Fig 5 also illustrates that simplification will likely not hold for pressure-sensitive proteins, with larger Δ*v*_un._

Those limitations notwithstanding, from the perspective of shelf life prediction and accelerated stability testing, an isochoric approach could provide interesting benefits of both high pressure and low temperature, in that both can shift conformational equilibria of proteins to help promote aggregation. The results here illustrate that protein preservation or storage stability, just like protein (un)folding equilibria, is not a monotonic function of temperature. To date, a relatively small number of proteins have been studied from the perspective of cold unfolding.[13,29–31] The phenomena of cold-unfolding and cold-aggregation appear to be intrinsically linked, and the results illustrate a viable experimental approach for that relationship to be explored more generally in future work.

## Conclusions

The results illustrate with BHb that the mechanisms that lead to aggregation in “hot” solutions can be relevant for “cold” solutions such as liquid below 0°C, where “cold” unfolding can help to drive aggregation. While the present case represents a simplified example in that high-pressure effects appear to be minimal for BHb, the more general case of isochoric cooling could offer a useful combination of cold- and pressure-induced unfolding to more readily accelerate aggregation rates for prediction of low-temperature storage of valuable proteins such as for biopharmaceuticals and other biotechnology applications.
